# Effect of income level on stroke incidence and the mediated effect of simultaneous diagnosis of metabolic syndrome diseases; a nationwide cohort study in South Korea

**DOI:** 10.1186/s13098-022-00882-1

**Published:** 2022-08-08

**Authors:** Seungmin Jeong, Sung-il Cho, So Yeon Kong

**Affiliations:** 1 Graduate School of Public Health, Seoul National University, 1 Gwanak-ro, Gwanak-gu, Seoul, 08826 Republic of Korea; 2 Department of Preventive Medicine, Kangwon National University Hospital, Chuncheon, Republic of Korea; 3 Gangwon Center for Infectious Diseases, Chuncheon, Republic of Korea; 4 Laerdal Medical, Stavanger, Norway

**Keywords:** Stroke, Metabolic syndrome, Socioeconomic factors, Healthcare disparities, Mediation analysis

## Abstract

**Background:**

This study aimed to determine whether a simultaneous diagnosis of main components of metabolic syndrome (MetS) (hypertension, diabetes mellitus, and dyslipidemia) plays a mediator between income level and stroke.

**Methods:**

We used the National Health Insurance Service National Sample Cohort database from 2006 to 2015. The mediator variables were the number of main MetS components diagnosed simultaneously (two or more/three or more). We used a weighting approach method of causal mediation analysis to apply counterfactual frameworks to the Cox proportional hazards regression model.

**Results:**

A total of 213,526 people were included with 1,690,665.3 person-years of followed up. Compared with the high-income group, the risk of being diagnosed with two or more components of MetS significantly increased in all other income groups [middle-income OR 1.05 (95% CI 1.02–1.08); low-income OR 1.09 (95% CI 1.05–1.12); Medical Aid beneficiaries OR 1.39 (95% CI 1.32–1.47)]. A lower level of income was significantly associated with a higher risk of stroke compared with the high-income group [middle-income HR 1.15 (95% CI 1.07–1.25); low-income HR 1.19 (95% CI 1.10–1.29); Medical Aid beneficiaries HR 1.63 (95% CI 1.48–1.80)]. In the Medical Aid beneficiaries, simultaneous diagnosis of the main metabolic components acted as a significant mediator between income levels and stroke incidence, with 26.6% mediated when diagnosed with two or more diseases and 21.1% when diagnosed with all three.

**Conclusions:**

Co-diagnosis of MetS components played a significant mediator role between income level and stroke incidence.

**Supplementary Information:**

The online version contains supplementary material available at 10.1186/s13098-022-00882-1.

## Background

Socioeconomic determinants are essential factors in the occurrence of stroke. Several previous studies have reported that low socioeconomic status, such as low income, increases the risk of stroke.

MetS (Metabolic syndrome) is a set of co-occurring diseases that increase the risk of cardiovascular disease [1]. MetS is known to increase the risk of stroke [2–4]. While there are several criteria for definitions of MetS, the most commonly used criteria used throughout the world include following components; abdominal obesity, insulin resistance, dyslipidemia, and hypertension [4]. These components are often diagnosed together, and they share mutual risk factors such as aging, obesity, and smoking [5]. It is controversial whether having two or more of these components at the same time increases the risk of stroke. Some studies reported that having those diseases simultaneously at the same time has little or no excess risk on stroke occurrence [6, 7]. On the other hand, other studies announced that simultaneous diagnosis of these diseases had a higher risk of stroke [5, 8].

Several previous studies reported that lower socioeconomic status increases the risk of MetS and its associated risk factors and diseases, such as hypertension, dyslipidemia, and type 2 diabetes [9–12]. While we consider the link between income level, diseases belonging to the MetS, and stroke, it is expected that the incidence of diseases belonging to the MetS plays as a mediator between income level and stroke. However, there have been no studies on this to date. Moreover, because the diseases belonging to MetS are often diagnosed concurrently, it is expected that the mediation rate may be higher if two or more diseases are diagnosed simultaneously.

This study aimed to determine whether a simultaneous diagnosis of hypertension, diabetes mellitus, and dyslipidemia, which belong to the diagnosis criteria of MetS, plays a mediator between income level and stroke.

## Methods

### Study setting

South Korea has a universal health care (UHC) system that covers all citizens. The UHC in South Korea is comprised of National Health Insurance (NHI) and Medical Aid.

NHI populations are divided into "Insured Employees" and "Insured Self-employed" [13]. Workers and employers of all workplaces are registered as representatives of households of the Insured Employees. The Insured Employee category also includes the representatives' dependents and immediate family, including siblings and parents from both sides of the family. The rest of the people who are not registered as the Insured Employee category are included in the Insured Self-employed category. The NHI receives monthly premiums from the representative of each household. Thus the representative of each household and family members who registered as dependents are considered to have the same income. The Insured Employee's dependents do not need to live together with the representative. Unlike Insured Employees, Insured Self-employed cannot register dependent of the household representative unless they live together. Therefore, the Insured Self-employed category reflects the individual's income level more accurately than the Insured Employee category [14]. For the Insured Self-employed category, monthly NHI premiums are determined by household income, wealth, standard of living, and economic activity participation rate. In addition, all NHI members pay a portion of the cost when using medical services designated by the NHI-approved Services. In South Korea, 57.7% of the total population is the Insured employee and 38.6% is the Insured Self-employed. Around 3.7% of the total population is Medical Aid beneficiaries [13].

Medical Aid is a public assistance program aimed at securing a minimum livelihood for low-income households by providing medical services nearly free [15]. Medical Aid beneficiaries do not pay health insurance premiums. Because the South Korean government pays expenses for their medical services, Medial Aid beneficiaries pay little or no cost for using NHI-approved medical services. [13]

### Data source

In this study, the National Health Insurance Service National Sample Cohort (NHIS-NSC) database of South Korea’s NHI Service from 2006 to 2015 was used [16]. This cohort database consists of a sample cohort of over one million people randomly selected from among 48,222,537 people of South Korea. This database provides each individual’s medical service usage history based on the subject's medical billing data from 2002 to 2015. In this study, we used the data from 2006 to 2015 because this dataset did not include the Medical Aid beneficiaries’ medical usage information from 2002 to2005.

### Study population

The subjects of this study were adults 18 years of age or older who were registered as Insured Self-employed and Medical Aid beneficiaries in the NHIS-NSC in 2008. As we mentioned in the study setting section, because the Insured Self-employed category reflects the individual's income level more accurately than the Insured Employee category, we excluded the Insured Employees and their dependents. Medical Aid beneficiaries generally have lower incomes than low-income households of the NHI. A mandatory requirement for Medical Aid is that all household members are not working. Thus, Medical Aid beneficiaries were included in the study as a group with a lower income than the low-income group of Insured Self-employed category.

We excluded the patients diagnosed with hypertension, diabetes mellitus, dyslipidemia, and stroke during the washout period (2006–2007). In addition, patients with a medical history of cardiovascular disease and cerebrovascular diseases such as transient ischemic attacks were excluded. Each disease was classified using 10th version of the International Classification of Diseases (ICD-10) diagnostic codes recorded in the medical record diagnosis. The ICD-10 codes for each disease were defined as below; I10-I15 for hypertension, E10-14 for diabetes mellitus, E78 for dyslipidemia, I60-I63 for stroke, I20-I25 for cardiovascular disease, and G45-46 for cerebrovascular disease [17]. We also excluded the cases that could not confirm the income level of individuals.

### Variables

The independent variable of this study was income level. We used the income level of 2008, based on the assumption that the income level in 2008 had not changed. In the NHIS-NSC, the income level of the Insured Self-employees is divided into deciles according to household income. Medical Aid beneficiaries are classified separately. In this study, income levels were classified into four groups; High-income (the top 1–3 decile in the Insured Self-employees), middle-income (the top 4–6 decile in the Insured Self-employees), low-income (the top 7–10 decile in the Insured Self-employees), and Medical Aid beneficiaries.

We validated the volatility of the income level to test the assumption that the income level at a specific point in time could represent the entire observation period. Among total 1,105,369 individuals in the NHIS-NSC dataset, we analyzed 514,148 people who belonged to the Insured Self-employed category/Medical Aid beneficiaries at least twice over a 10-year period from 2006 to 2015. The income level variability was confirmed as the standard deviation using this study's classification criteria, setting the high-income level as 1, the middle-income level as 2, the low-income level as 3, and Medical Aid beneficiaries as 4 as continuous variables. There was no change in the income level in 276,633 people (53.6% of the subjects), and the 75% quartile of the standard deviation was 0.52 with the maximum value of 2.12 (Additional file 2: Fig. S1). Considering the variability identified above, we were able to assume that for most of the study subjects the income level classification at a specific point in time did not show large fluctuations during the follow-up period, thus we concluded that the income level in the first year of the study could be used as an exposure variable.

The dependent variable was the occurrence of stroke.

The mediator variables were number of main MetS components (hypertension, diabetes mellitus, and dyslipidemia) diagnosed simultaneously (two or more/three or more). There are several criteria for the clinical diagnosis of MetS. The criteria most commonly used is the definition developed by the American Heart Association. In this definition, MetS is diagnosed when more than three of below five constitutes are satisfied; elevated waist circumference, elevated triglycerides, reduced HDL-C, elevated blood pressure, and elevated fasting glucose [18]. We regarded the three components—hypertension, diabetes mellitus, and dyslipidemia—as “MetS diseases.”

In addition, gender, age, region of residence, degree of disability, comorbidity, diagnosis of cardiovascular disease, and diagnosis of cerebrovascular disease such as transient ischemic attack were considered for confounding variables. Age was adjusted as a continuous variable. Residential areas were classified into four categories based on each individual’s residential area in 2008, those living: (1) Seoul, (2) metropolitan cities, (3) Gyeonggi-do (nearby Seoul), and (4) other provinces. The degree of physical disability was classified as severe and moderate, mild, and no disability. For comorbidities, the Charlson Comorbidity Index (CCI) was used. CCI was measured using the ICD codes of medical service records diagnosis in 2006–2007. [19]

### Statistical analysis

Baseline characteristics were described with frequency and percentage.

A multivariate logistic regression model was used to verify the association between income level (the independent variable) and the number of MetS diseases diagnosed simultaneously (the mediator variable).

Traditional mediation analysis had calculated the proportion of mediating effects by comparing the estimates of the model without a mediator and the model with the mediator [20]. However, previous studies have found that the traditional approach may overestimate or underestimate the actual effects of mediators because of the below reasons: (1) mediator-outcome confounding, (2) exposure-mediator interaction, and (3) mediator-outcome confounding affected by the exposure [21–23]. Therefore, we used a causal mediation analysis method based on a counterfactual framework to overcome these flaws. In this study, survival analysis was performed using the Cox proportional hazards regression model. In order to apply the counterfactual framework to this model, a weighting approach method was used [24].

In each mediation analysis, the mediation proportion was obtained by modifying the equation used by VanderWeele and Vansteelandt, replacing an odds ratio to a hazard ratio [25].

$$\frac{{\mathrm{aHR}}^{NDE} ({aHR}^{NIE}-1)}{(a{HR}^{NDE} \times a{HR}^{NIE}-1)}$$

where, aHR: adjusted Hazard Ratio, NDE: Natural Direct Effect, NIE: Natural Indirect Effect.

Analysis was performed using SAS Enterprise Guide version 7.1(SAS Institute Inc., Cary, NC, USA). In the logistic regression analysis and survival analysis, confounding variables such as sex, age, region of residence, degree of disability, CCI, and diagnosis of cardiovascular disease and cerebrovascular disease were adjusted.

### Ethics statement

This study was approved by the Institutional Review Board of Seoul National University (IRB No. E1808/003-002).

## Results

In the NHIS-NSC, adults 18 years of age or older in 2008 were 807,162. We excluded 27,931 people diagnosed with stroke before 2008 and 252,078 people diagnosed with hypertension, diabetes mellitus, dyslipidemia, cardiovascular disease, or cerebrovascular disease before 2008. We also excluded 313,553 people in the Insured Employee category and 74 persons whose income decile could not be verified. Finally, 213,526 people were enrolled, and a total of 1,690,665.3 person-years were followed up.

Table 1 shows the demographics of the study population by income level. The number of the diagnosed MetS diseases was different by income level. In the high-income group, 62.5% were not diagnosed with any of the MetS diseases, and 4.4% with all three of the MetS diseases. In the Medical Aid beneficiaries, 49.0% were not diagnosed with any of the MetS diseases, and 10.4% with all three of MetS diseases. Overall, among the MetS diseases, the diagnosis rate of dyslipidemia was the highest (29.2%). The stroke incidence rate was 1.7% in the high-income group, 1.9% in the middle-income group, 2.1% in the low-income group, and 6.3% in the Medical Aid beneficiaries.

**Table 1:** Demographics of the study population

	Total		High Income		Middle Income		Low Income		Medical aid beneficiaries		*p*-value
	N	(%)	N	(%)	N	(%)	N	(%)	N	(%)	
Total	213,526	100.0	75,824	100.0	67,986	100.0	57,587	100.0	12,129	100.0	
Sex											< 0.01
Male	109,520	51.3	38,193	50.4	35,781	52.6	29,922	52.0	5624	46.4	
Female	104,006	48.7	37,631	49.6	32,205	47.4	27,665	48.0	6505	53.6	
Age											< 0.01
18–39 years old	112,477	52.7	38,856	51.2	36,859	54.2	31,421	54.6	5341	44.0	
40–59 years old	87,011	40.7	32,380	42.7	27,615	40.6	22,435	39.0	4581	37.8	
≥ 60 years old	14,038	6.6	4588	6.1	3512	5.2	3731	6.5	2207	18.2	
Residential area											< 0.01
Seoul	46,479	21.8	18,437	24.3	13,547	19.9	12,887	22.4	1608	13.3	
Metropolitan	55,146	25.8	18,082	23.8	18,843	27.7	14,785	25.7	3436	28.3	
Gyeongi (near Seoul)	46,648	21.8	19,376	25.6	13,980	20.6	11,722	20.4	1570	12.9	
Other	65,253	30.6	19,929	26.3	21,616	31.8	18,193	31.6	5515	45.5	
CCI											< 0.01
0	152,769	71.5	53,448	70.5	49,286	72.5	42,998	74.7	7037	58.0	
1	45,286	21.2	16,948	22.4	14,276	21.0	11,037	19.2	3025	24.9	
2 and more	15,471	7.2	5428	7.2	4424	6.5	3552	6.2	2067	17.0	
Physical disability severity											< 0.01
None	204,796	95.9	74,312	98.0	66,308	97.5	55,322	96.1	8854	73.0	
Mild	2745	1.3	307	0.4	320	0.5	343	0.6	1775	14.6	
Moderate and severe	5985	2.8	1205	1.6	1358	2.0	1922	3.3	1500	12.4	
Diagnosis of IHD											< 0.01
Yes	11,219	5.3	3858	5.1	3300	4.9	2934	5.1	1127	9.3	
Diagnosis of CVD (except stroke)											< 0.01
Yes	2975	1.4	1010	1.3	878	1.3	726	1.3	361	3.0	
Number of diagnosis of MetS disease											< 0.01
0	132,969	62.3	47,425	62.5	43,270	63.6	36,336	63.1	5938	49.0	
1	45,916	21.5	16,722	22.1	14,330	21.1	11,956	20.8	2908	24.0	
2	23,992	11.2	8318	11.0	7343	10.8	6313	11.0	2018	16.6	
3	10,649	5.0	3359	4.4	3043	4.5	2982	5.2	1265	10.4	
Diagnosis of HTN											< 0.01
Yes	31,422	14.7	10,406	13.7	9581	14.1	8646	15.0	2789	23.0	
Diagnosis of DM											< 0.01
Yes	27,464	12.9	9,082	12.0	8260	12.1	7479	13.0	2643	21.8	
Diagnosis of dyslipidemia											< 0.01
Yes	62,388	29.2	22,650	29.9	18,982	27.9	16,152	28.0	4604	38.0	
Stroke incidence											< 0.01
Yes	4589	2.1	1304	1.7	1281	1.9	1236	2.1	768	6.3	

*CCI* Charlson Comorbidity Index, *IHD* ischemic heart disease, *CVD* cardiovascular disease, *MetS* metabolic syndrome; *HTN* hypertension, *DM* diabetes mellitus

Table 2 shows the multivariate logistic regression analysis results on the risk of a simultaneous diagnosis of MetS diseases varies by income level. Compared with the high-income group, the risk of being diagnosed with two or more MetS diseases significantly increased in all other income groups. The aOR (adjusted odds ratio) for the middle-income group was 1.05 (95% CI 1.02–1.08), and the aOR for the low-income group was 1.09 (95% CI 1.05–1.12). The aOR for Medical Aid beneficiaries was 1.39 (95% CI 1.32–1.47). The risk of being diagnosed with all three MetS diseases was significantly higher in all income groups compared with the high-income group. The aOR was 1.08 (95% CI 1.03–1.14) for the middle-income group, 1.20 (95% CI 1.14–1.27) for the low-income group, and 1.50 (95% CI 1.38–1.63) for Medical Aid beneficiaries.

**Table 2:** Results of multivariate logistic regression analysis on the risk of a simultaneous diagnosis of MetS diseases by income level

	Total (n)	Diagnosis of disease(s) (n)	Rate (%)	Adjusted OR (95% CI)
Simultaneous diagnosis of two or more MetS diseases				
High income	75,824	11,677	15.4	Reference
Middle income	67,986	10,386	15.3	1.05 (1.02–1.08)
Low income	57,587	9295	16.1	1.09 (1.05–1.12)
Medical aid beneficiaries	12,129	3283	27.1	1.39 (1.32–1.47)
Simultaneous diagnosis of three MetS diseases				
High income	75,824	3359	4.4	Reference
Middle income	67,986	3043	4.5	1.08 (1.03–1.14)
Low income	57,587	2982	5.2	1.20 (1.14–1.27)
Medical aid beneficiaries	12,129	1265	10.4	1.50 (1.38–1.63)

*MetS* metabolic syndrome, *OR* odds ratio, *CI* confidence interval

MetS diseases: hypertension, diabetes mellitus, dyslipidemia

Table 3 shows survival analysis results using the Cox proportional hazards model for stroke incidence according to the income level. Model 1 is the result of adjusting for age, sex, residential area, CCI, disability severity, and diagnosis of cardiovascular and cerebrovascular diseases. Model 2 results were obtained by adding adjustment of the simultaneous diagnosis of two or more MetS diseases to Model 1. In Model 2, all income groups had a significantly higher risk of stroke than the high-income group. The middle-income group had 1.15 times (95% CI 1.07–1.25) higher risk of stroke than the high-income group, and the low-income group had 1.19 times (95% CI 1.10–1.29), and Medical Aid beneficiaries had 1.63 times (95% CI 1.48–1.80). The HR (hazard ratio) for the simultaneous diagnosis of two or more MetS diseases was 5.44 (95% CI 5.10–5.80). Model 3 adds adjustment of the simultaneous diagnosis of all three MetS diseases to Model 1. All of the HRs of income levels decreased in Model 3 compared to Models 1 and 2. It was 1.14 (95% CI 1.05–1.23) for the middle-income group, 1.17 (95% CI 1.08–1.26) for the low-income group, and 1.61 (95% CI 1.46–1.78) for Medical Aid beneficiaries. The HR for the simultaneous diagnosis of all three MetS diseases was 4.50 (95% CI 4.21–4.81).

**Table 3:** Results of the survival analysis for stroke incidence by income level

	Total(n)	Stroke incidence (n)	Rate (%)	Adjusted HR model 1 (95% CI)	Adjusted HR model 2 (95% CI)	Adjusted HR model 3 (95% CI)
High income	75,824	1304	1.7	Reference	Reference	Reference
Middle income	67,986	1281	1.9	1.16 (1.07–1.25)	1.15 (1.07–1.25)	1.14 (1.05–1.23)
Low income	57,587	1236	2.1	1.21 (1.12–1.31)	1.19 (1.10–1.29)	1.17 (1.08–1.26)
Medical aid beneficiaries	12,129	768	6.3	1.75 (1.59–1.93)	1.63 (1.48–1.80)	1.61 (1.46–1.78)
Simultaneous diagnosis of two or more MetS diseases				–	5.44 (5.10–5.80)	–
Simultaneous diagnosis of three MetS diseases				–	–	4.50 (4.21–4.81)

MetS diseases: hypertension, diabetes mellitus, dyslipidemia

Model 1: adjusted for age, sex, residential area, Charlson Comorbidity Index, physical disability severity, diagnosis of ischemic heart disease, and diagnosis of cerebrovascular disease (except stroke)

Model 2: adjusted for age, sex, residential area, Charlson Comorbidity Index, physical disability severity, diagnosis of ischemic heart disease, diagnosis of cerebrovascular disease(except stroke), and simultaneous diagnosis of MetS diseases (≥ 2)

Model 3: adjusted for age, sex, residential area, Charlson Comorbidity Index, physical disability severity, diagnosis of ischemic heart disease, diagnosis of cerebrovascular disease(except stroke), and simultaneous diagnosis of MetS diseases (= 3)

MetS metabolic syndrome, *HR* hazard ratio, *CI* confidence interval

**Fig. 1:**
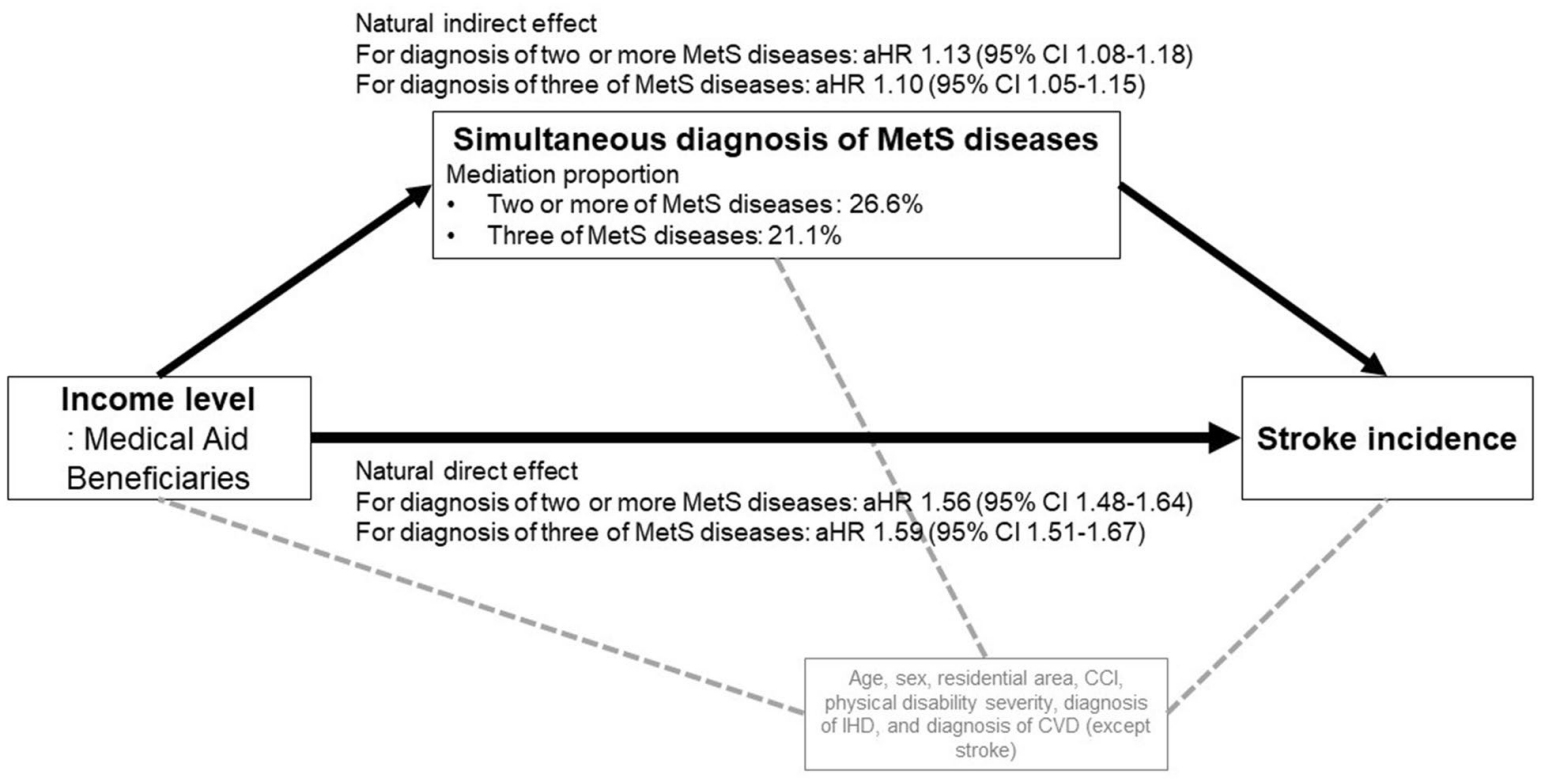
The mediation effect of the simultaneous diagnosis of MetS diseases between the Medical Aid beneficiaries and the stroke incidence. *MetS* metabolic syndrome, *aHR* adjusted hazard ratio, *CI* confidence interval, *CCI* Charlson comorbidity index, *IHD* Ischemic heart disease, *CVD* cerebrovascular disease. MetS diseases: hypertension, diabetes mellitus, dyslipidemia

Table 4 shows the results of the weighted approach mediation analysis based on the counterfactual framework. It was found that the natural indirect effects of a simultaneous diagnosis of MetS diseases were significant only for Medical Aid beneficiaries. When the diagnosis of two or more MetS diseases was a mediator, the mediation rate was 26.6%, and when the diagnosis of three MetS diseases was a mediator, the mediation rate was 21.1% (Fig. 1).

**Table 4:** Results of the weighted approach mediation analysis based on the counterfactual framework

Income level	Model A		Model B	
	Adjusted HR (95% CI)	Mediation proportion (%)	Adjusted HR (95% CI)	Mediation proportion (%)
High income	Reference		Reference	
Middle income: natural direct effect	1.14 (1.09–1.18)		1.13 (1.09–1.18)	
Low income: natural direct effect	1.18 (1.13–1.22)		1.16 (1.11–1.21)	
Medical Aid beneficiaries: natural direct effect	1.56 (1.48–1.64)		1.59 (1.51–1.67)	
Middle income: natural indirect effect	1.02 (0.98–1.06)		1.02 (0.98–1.06)	
Low income: natural indirect effect	1.03 (0.99–1.07)		1.04 (1.00–1.08)	
Medical Aid beneficiaries: natural indirect effect	1.13 (1.08–1.18)	26.6	1.10 (1.05–1.15)	21.1

Model A: for diagnosis of two or more Metabolic Syndrome diseases

Model B: for diagnosis of three Metabolic Syndrome diseases

Each model was adjusted for age, sex, residential area, Charlson Comorbidity Index, physical disability status, diagnosis of ischemic heart disease, and diagnosis of cerebrovascular disease (except stroke)

*HR* hazard ratio, *CI* confidence interval

## Discussion

In this study, the lower-income groups had a higher risk of a simultaneous diagnosis of MetS diseases than the high-income group. The risk of simultaneous diagnosis of MetS diseases was significantly higher in all the other income groups than in the high-income group. Also, as the number of diagnosed diseases increased, the hazard risks also increased. In addition, the risk of stroke incidence was significantly higher in all the other income groups than in the high-income group. Simultaneous diagnosis of MetS diseases was a significant mediator between income level and stroke occurrence in the Medical Aid beneficiaries. The mediation proportion was 26.6% when the diagnosis of two or more MetS diseases was a mediator. In the case of diagnosis of three MetS diseases, the mediation proportion was 21.1%.

In previous studies, non-communicable diseases, especially diseases included in diagnostic criteria for metabolic syndrome such as hypertension, diabetes mellitus, and dyslipidemia, were closely related to income levels [9–12]. There was also a close association between income level and stroke incidence [14, 26–28]. However, there were no studies on the mediating effect of a simultaneous diagnosis of MetS diseases between income level and stroke incidence. This study found that co-diagnosis of major diseases belonging to MetS diagnostic criteria plays a significant mediator role between income level and stroke incidence with high mediation proportions (26.6% for two or more and 21.1% for three MetS diseases) in the Medical Aid beneficiaries group.

In this study, we did not directly use the diagnostic definition of MetS proposed by the American Heart Association in 2005. Instead, for the mediator variable, we made a new definition of "MetS diseases" and defined a mediator as two or three of "MetS diseases" are diagnosed simultaneously. The NHIS-NSC provides the subjects' national health check-up results, and abdominal obesity is a component of the health check-up list. However, using the health check-up data is concerned about serious selection bias because national health check-up is not mandatory, and the health check-up rate rapidly decreases as income decreases [29]. When we checked the proportion of the health check-up by the income level with this dataset, it was confirmed that the proportion of people who received a health check-up before diagnosing a stroke decreased according to their income level (the high-income group: 63.5%, the middle-income group: 60.7%, the low-income group: 54.1%, and Medical Aid beneficiaries: 46.7%). Also, since it is a one-time examination, the figures may be temporarily inaccurate. Therefore, the health check-up results were not used for this analysis, and the medical record of outpatient and inpatient treatment were used. In general, abdominal obesity does not require outpatient and inpatient treatment. For this reason, abdominal obesity was not included in the elements for the "MetS diseases." However, we assume that using this definition would be similar to using an exact definition of metabolic syndrome. Because, first, two of the five diagnostic criteria for MetS are diagnostic criteria for dyslipidemia. Furthermore, in the sensitivity analysis performed using health check-up data, there was no significant difference between the results using the definition used in this study and the result of adding abdominal obesity for an element (Additional file 1: Tables S1–S5). Therefore, we believe that the results of this study provide evidence that MetS has a mediating effect between income level and stroke.

In our study, the mediating effect of the simultaneous diagnosis of MetS diseases was not significant in the middle- and low-income groups; however, it was significant in the Medical Aid beneficiaries group. We also found the higher risk of both simultaneous diagnosis of MetS diseases and stroke in the Medical Aid beneficiaries group than other income groups. Since MetS diseases are multifaceted health problems, they cannot be treated with a single agent. Therefore, the management of MetS diseases cannot be resolved with medical expense support alone, but should be accompanied by multiple interventions, including lifestyle modification, high medication compliance, and relieving mental stress, which is one of the main challenges particularly for Medical Aid beneficiaries [9, 30–32]. This relationship among the independent variable, the mediator variable, and the outcome variable would be a reason that the mediating effect of the Medical Aid beneficiaries was significant with a high proportion compared to other income groups. Furthermore, in a previous study, Medical Aid beneficiaries had more outpatient visits than NHI members, and there was no difference in the number of outpatient visits according to income within the Medical Aid beneficiaries; because they receive treatment of diseases such as hypertension, diabetes mellitus, or dyslipidemia treatment for free or near to free [33]. Thus, there are low chances of misclassifying a MetS disease patient as a nonpatient in the Medical Aid beneficiaries group, which may have led more accurate measure of the mediating effect in the Medical Aid beneficiaries group in this study.

Considering these results, to prevent inequity in stroke occurrence, it is necessary to prevent hypertension, diabetes mellitus, and dyslipidemia, especially among Medical Aid beneficiaries. Management of health behaviors such as smoking cessation, abstinence of binge drinking, encouragement of physical activity, and mental stress management are essential [34]. Management of newly diagnosed patients with MetS will also be critical. Medication management and removal of risk factors that aggravate the disease are required.

The mediating effect was significant only in the Medical Aid beneficiaries. However, all the other income groups had a higher risk than the high-income group to diagnose MetS diseases simultaneously, and the magnitude of the hazard ratios increased as the income level decreased. The same trend was observed for the occurrence of stroke, which suggests that income inequity for diagnosis of MetS and stroke has a gradient pattern, and the health of all the population is affected by the inequity. It provides evidence that policies for health inequity should focus on the entire population, not just the most disadvantaged population [35].

The finding that income level acts as a significant risk factor for poor health outcomes means that this is not a matter of individual will but a social context that makes this prevention challenging for the lower-income class. For the lower-income patients, unhealthy health behaviors such as smoking, drinking alcohol, poor eating habits, and lack of physical activity often increase the risk of metabolic syndrome and stroke [34]. In previous studies, people with a low income have a tendency to focus more on the present situation than on long-term investments in future life. They also had a higher possibility of choosing unhealthy behavior because of the opportunity cost of obtaining good health behavior, pessimism about the future, and the influence of family and neighbors with similar environments [36]. Therefore, there is a need for a policy that goes beyond simply carrying out health behavior promotion projects and makes it easier for people with low incomes to choose healthy habits.

In this study, the mediation rate was the highest when two or more MetS diseases were diagnosed than when all three were diagnosed. When we performed an additional analysis using the diagnosis of one of the three diseases as a mediator, the mediating effect was significant in Medical Aid beneficiaries, and the mediation rate was 19.5% (Additional file 1: Table S6). We can explain it below: In the mediation analysis, the mediation rate is higher when: (1) the greater the effect of the mediator on the outcome, and (2) the greater proportion of the population with the mediator. In this study we observed: (1) the greater the number of diagnosed MetS diseases, the more significant contribution they had on the occurrence of stroke (outcome), and (2) the proportion of the patients with the mediator decreased as the number of the diagnosed MetS diseases increased. As shown in Table 3, as the number of diagnosed MetS diseases increased, the risk of income level on stroke decreased. Therefore, though the mediation rate was higher when two or more MetS diseases were diagnosed, more attention should be paid to people with more diseases(those with 3 MetS diseases) when applying it in actual policy.

As mentioned in the Methods section, we tried to avoid underestimating or overestimating mediating effects in this study by applying the causal mediation analysis method. We compared our result and the result of the traditional mediation analysis by performing additional analysis. In the traditional mediation analysis, we found that in both cases- the mediator was a diagnosis of two or more MetS diseases, or all three MetS diseases- mediated effects were significant in all the other income groups compared with the high-income group. When applying traditional mediation analysis, the magnitudes of mediating effects in Medical Aid beneficiaries were higher than when calculated through causal mediation analysis (Additional file 1: Table S7).

This study has several strengths. First, the mediating effect was measured more accurately using the causal mediation analysis, overcoming the weaknesses of the traditional mediation analysis. Second, the data we used can represent the entire country’s population. Third, as only 3599 people, or 1.7% of the total enrolled population, were censored before 2015, the last year, the completeness of the data is excellent.

This study has several limitations. First, we classified the income level by the income level in 2008, assuming that the income would not change. Thus there is a possibility of errors due to the change in income. However, as we described in the Method section, in the results of testing the income level volatility, we found that most people did not experience a change or experienced subtle changes in their income level during a 10-year period. Thus, we assume that the bias from the change in the income level is minimal. Second, there is an opportunity for a difference between the diagnosis and the actual prevalence because the case of diagnosis was confirmed based on the medical usage record. Third, since the duration of having the Mets diseases was not reflected, the effect of the accumulation of diseases could not be reflected. Fourth, there is a possibility of unmeasured confounding effects, such as pharmacological treatment status of the patients (drug interactions). In addition, we did not use the official diagnostic criteria for MetS directly.

## Conclusions

In this study, low income was a significant risk factor for simultaneous diagnosis of main MetS components and stroke. Co-diagnosis of MetS components played a significant mediator role between income level and stroke incidence. Therefore, there is a need for a policy to prevent and manage metabolic syndrome, especially for low-income patients.

## Supplementary Information

**Additional file 1:** **Table S1.** Results with the definition used in this study as a mediator (diagnosis of two or more MetS diseases) in the health check-up data. **Table S2.** Results with the new definition as a mediator (diagnosis of two or more among "hypertension, diabetes mellitus, dyslipidemia, and abdominal obesity") in the health check-up data. **Table S3.** Results with the definition used in this study as a mediator (diagnosis of three of MetS diseases) in the health check-up data. **Table S4.** Results with the new definition as a mediator (diagnosis three or more among "hypertension, diabetes mellitus, dyslipidemia, and abdominal obesity") in the health check-up data. **Table S5.** Results with the new definition as a mediator (diagnosis of four among "hypertension, diabetes mellitus, dyslipidemia, and abdominal obesity") in the health check-up data. **Table S6.** Causal mediation analysis result: the mediator was a diagnosis of one or more MetS diseases. **Table S7.** Result of the traditional mediation analysis.

**Additional file 2:** **Fig. S1.** The volatility of the income level in 514,148 subjects in the dataset. X-axis: Standard deviation of the changes in the income level in each individual. Y-axis: % of the subjects.

## Data Availability

The data used in this study are provided by the National Health Insurance Service(NHIS). Any researcher who wishes to access the dataset should obtain permission from the NHIS and pay of appropriate license fee.

## Abbreviations

MetS: Metabolic syndrome
UHC: Universal health care
NHI: National Health Insurance
NHIS-NSC: National Health Insurance Service National Sample Cohort
ICD-10: 10Th version of the International Classification of Diseases
CCI: Charlson comorbidity index
aOR: Adjusted odds ratio
