# Supplementary material for: Effect of income level on stroke incidence and the mediated effect of simultaneous diagnosis of metabolic syndrome diseases; a nationwide cohort study in South Korea
Source: Diabetol Metab Syndr. 2022 Aug 8;14:110. doi: 10.1186/s13098-022-00882-1 (PMC9358809; doi:10.1186/s13098-022-00882-1)
Supplement: Supplementary file 1 — Additional file 1: Table S1. Results with the definition used in this study as a mediator (diagnosis of two or more MetS diseases) in the health check-up data. Table S2. Results with the new definition as a mediator (diagnosis of two or more among "hypertension, diabetes mellitus, dyslipidemia, and abdominal obesity") in the health check-up data. Table S3. Results with the definition used in this study as a mediator (diagnosis of three of MetS diseases) in the health check-up data. Table S4. Results with the new definition as a mediator (diagnosis three or more among "hypertension, diabetes mellitus, dyslipidemia, and abdominal obesity") in the health check-up data. Table S5. Results with the new definition as a mediator (diagnosis of four among "hypertension, diabetes mellitus, dyslipidemia, and abdominal obesity") in the health check-up data. Table S6. Causal mediation analysis result: the mediator was a diagnosis of one or more MetS diseases. Table S7. Result of the traditional mediation analysis. [file 13098_2022_882_MOESM1_ESM.docx]

Additional file 1

Table S1. Results with the definition used in this study as a mediator (diagnosis of two or more MetS diseases) in the health check-up data

| Income level | | Adjusted HR  (95% CI) | Mediation  proportion (%) |
| --- | --- | --- | --- |
|  | High income | Reference |  |
|  | Middle income: natural direct effect | 1.14 (1.08-1.19) |  |
|  | Low income: natural direct effect | 1.18 (1.13-1.24) |  |
|  | Medical Aid beneficiaries: natural direct effect | 1.50 (1.38-1.62) |  |
|  | Middle income: natural indirect effect | 0.97 (0.97-1.08) |  |
|  | Low income: natural indirect effect | 0.99 (1.99-1.10) |  |
|  | Medical Aid beneficiaries: natural indirect effect | 1.16 (1.10-1.22) | 32.0 |

MetS=Metabolic Syndrome; HR=hazard ratio; CI=confidence interval.

Table S2. Results with the new definition as a mediator (diagnosis of two or more among "hypertension, diabetes mellitus, dyslipidemia, and abdominal obesity") in the health check-up data

| Income level | | Adjusted HR  (95% CI) | Mediation  proportion (%) |
| --- | --- | --- | --- |
|  | High income | Reference |  |
|  | Middle income: natural direct effect | 1.14 (1.09-1.2) |  |
|  | Low income: natural direct effect | 1.2 (1.14-1.26) |  |
|  | Medical Aid beneficiaries: natural direct effect | 1.53 (1.51-1.66) |  |
|  | Middle income: natural indirect effect | 1.02 (0.97-1.08) |  |
|  | Low income: natural indirect effect | 1.03 (0.98-1.09) |  |
|  | Medical Aid beneficiaries: natural indirect effect | 1.13 (1.07-1.19) | 27.2 |

HR=hazard ratio; CI=confidence interval.

Table S3. Results with the definition used in this study as a mediator (diagnosis of three of MetS diseases) in the health check-up data

| Income level | | Adjusted HR  (95% CI) | Mediation  proportion (%) |
| --- | --- | --- | --- |
|  | High income | Reference |  |
|  | Middle income: natural direct effect | 1.13 (1.08-1.19) |  |
|  | Low income: natural direct effect | 1.18 (1.12-1.24) |  |
|  | Medical Aid beneficiaries: natural direct effect | 1.53 (1.41-1.66) |  |
|  | Middle income: natural indirect effect | 1.02 (0.97-1.08) |  |
|  | Low income: natural indirect effect | 1.04 (0.99-1.10) |  |
|  | Medical Aid beneficiaries: natural indirect effect | 1.13 (1.07-1.19) | 27.4 |

MetS=Metabolic Syndrome; HR=hazard ratio; CI=confidence interval.

Table S4. Results with the new definition as a mediator (diagnosis three or more among "hypertension, diabetes mellitus, dyslipidemia, and abdominal obesity") in the health check-up data

| Income level | | Adjusted HR  (95% CI) | Mediation  proportion (%) |
| --- | --- | --- | --- |
|  | High income | Reference |  |
|  | Middle income: natural direct effect | 1.13 (1.08-1.19) |  |
|  | Low income: natural direct effect | 1.18 (1.13-1.24) |  |
|  | Medical Aid beneficiaries: natural direct effect | 1.54 (1.42-1.67) |  |
|  | Middle income: natural indirect effect | 1.02 (0.97-1.08) |  |
|  | Low income: natural indirect effect | 1.04 (0.99-1.10) |  |
|  | Medical Aid beneficiaries: natural indirect effect | 1.13 (1.07-1.19) | 27.1 |

HR=hazard ratio; CI=confidence interval.

Table S5. Results with the new definition as a mediator (diagnosis of four among "hypertension, diabetes mellitus, dyslipidemia, and abdominal obesity") in the health check-up data

| Income level | | Adjusted HR  (95% CI) |
| --- | --- | --- |
|  | High income | Reference |
|  | Middle income: natural direct effect | 1.16 (1.1-1.21) |
|  | Low income: natural direct effect | 1.22 (1.16-1.29) |
|  | Medical Aid beneficiaries: natural direct effect | 1.64 (1.51-1.78) |
|  | Middle income: natural indirect effect | 1.01 (0.95-1.06) |
|  | Low income: natural indirect effect | 1.01 (0.96-1.07) |
|  | Medical Aid beneficiaries: natural indirect effect | 1.04 (0.98-1.10) |

HR=hazard ratio; CI=confidence interval.

Table S6. Causal mediation analysis result: the mediator was a diagnosis of one or more MetS diseases

| Income level | | Adjusted HR  (95% CI) | Mediation  proportion (%) |
| --- | --- | --- | --- |
|  | High income | Reference |  |
|  | Middle income: natural direct effect | 1.16 (1.12-1.21) |  |
|  | Low income: natural direct effect | 1.22 (1.17-1.27) |  |
|  | Medical Aid beneficiaries: natural direct effect | 1.62 (1.54-1.71) |  |
|  | Middle income: natural indirect effect | 0.99 (0.95-1.04) |  |
|  | Low income: natural indirect effect | 1,00 (0.96-1.04) |  |
|  | Medical Aid beneficiaries: natural indirect effect | 1.09 (1.05-1.14) | 19.5 |

MetS=Metabolic Syndrome; HR=hazard ratio; CI=confidence interval.

Table S7. Result of the traditional mediation analysis

|  |  | Sobel test p-value | Indirect effect of exposure  mediated by mediator | Direct effect of exposure | Total effect (direct+indirect)  of exposure | Total effect (ignoring mediator)  of exposure | Proportion of  total effect mediated | Ratio of  indirect to direct |
| --- | --- | --- | --- | --- | --- | --- | --- | --- |
| Simultaneous diagnosis of two or more MetS diseases | | |  |  |  |  |  |  |
|  | Middle income | <0.01 | 0.09 | 0.14 | 0.23 | 0.14 | 0.37 | 0.6 |
|  | Low income | <0.01 | 0.14 | 0.18 | 0.31 | 0.19 | 0.44 | 0.8 |
|  | Medical Aid beneficiaries | <0.01 | 0.58 | 0.49 | 1.06 | 0.56 | 0.54 | 1.18 |
| Simultaneous diagnosis of three MetS diseases | | |  |  |  |  |  |  |
|  | Middle income | <0.01 | 0.12 | 0.13 | 0.25 | 0.14 | 0.49 | 0.94 |
|  | Low income | <0.01 | 0.28 | 0.15 | 0.43 | 0.19 | 0.64 | 1.79 |
|  | Medical Aid beneficiaries | <0.01 | 0.63 | 0.48 | 1.10 | 0.56 | 0.57 | 1.31 |

MetS=Metabolic Syndrome.
