# Supplementary figures and images for: Effect of income level on stroke incidence and the mediated effect of simultaneous diagnosis of metabolic syndrome diseases; a nationwide cohort study in South Korea
Source: Diabetol Metab Syndr. 2022 Aug 8;14:110. doi: 10.1186/s13098-022-00882-1 (PMC9358809; doi:10.1186/s13098-022-00882-1)

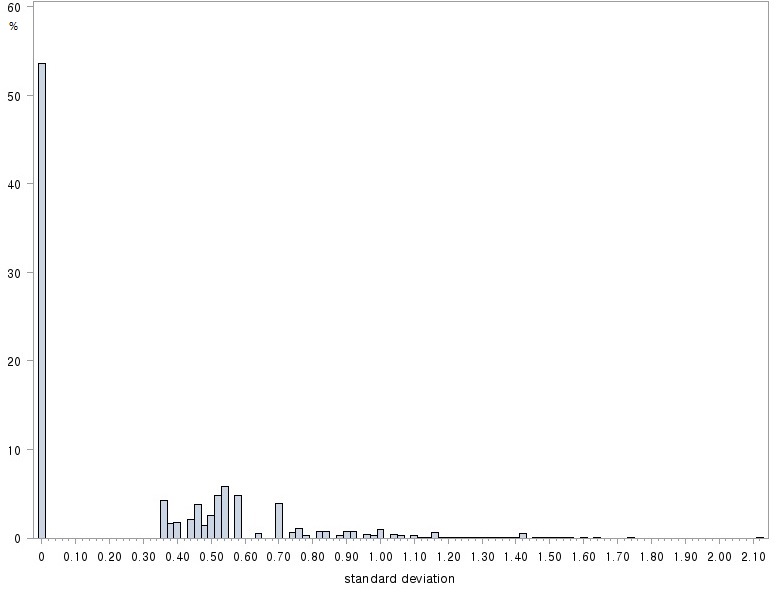

Supplement: Supplementary file 2 — Additional file 2: Fig. S1. The volatility of the income level in 514,148 subjects in the dataset. X-axis: Standard deviation of the changes in the income level in each individual. Y-axis: % of the subjects. [file 13098_2022_882_MOESM2_ESM.jpg]
